# Three Cases of Intermittent Fever and How They Were Treated

**Published:** 1894-04

**Authors:** C. M. Boger

**Affiliations:** Parkersburg, W. Va.


### THREE CASES OF INTERMITTENT FEVER AND HOW THEY WERE TREATED.

C. M. Boger, M. D., Parkersburg, W. Va.

### Case I.

September 17th, 1892.—Was called to see Mr. O. M. B., æt. fifty-nine. Two years ago patient moved here from the Wabash region of Indiana, where he suffered for sixteen years with attacks of ague. His present condition is as follows:

Very much emaciated, unable to leave house. Frequent undigested stools, provoked by eating and motion; continual ringing in ears; has abused Quinine for many years; chill every other day, beginning in occiput and spreading down spine, with no thirst; chill is followed by internal heat with thirst, but as soon as he drinks the external heat sets in, which is intense and causes him to sleep profoundly; during this sleep he desires to be uncovered; time of chill irregular; bitter taste in morning; nasal catarrh, with much purulent mucus from nose; desire to go to fire during chill, but gets no relief therefrom; sweat absent, or sometimes night-sweats; for this condition he received Chin.$^{200}$, three powders, then Sac-lac., and up to several weeks ago the chill had not returned in one year and one month. That looks like a cure. Whether the patient was afflicted with chronic cinchonism or ague, I have not made up my mind.

### Case II.

Mr. L. B., æt. sixty-six, attacked February 22d, 1893, with congestive headache, beginning with throbbing in the tonsils and occiput, less severe in other parts of head; with the headache there is a sense of hardness of the eyeballs and misty vision; also stiffness of the muscles of the neck, and dry mouth and lips. For this he received Bell.$^{3x}$, which gradually removed all the above symptoms, so that on February 27th he was able to come to the office, but reports the following state of affairs now, which is the return of an old complaint: Chill every other day at 11 A. M.; during chill has pulsation over whole body; this is followed by an internal heat and then a clammy skin, but no sweat. *Nat-mur.*$^{200}$ and Sac-lac. removed these remaining symptoms, since which he has been well.

### Case III.

October 15th, 1892.—Mr. F. P., æt. about forty: Chill sometimes at 11 A. M., at others at 2 P. M., commingled with heat; located mostly in the back, not relieved by any form of heat; during chill aching in calves of legs and back; heat in afternoon with burning in soles and eyes; absence of thirst in all stages. ℞.—Sul.$^{200}$, three doses, then Sac-lac., cured this case, there being no return in over a year.

